# Indicators of the relative availability of healthy versus unhealthy foods in supermarkets: a validation study

**DOI:** 10.1186/s12966-017-0512-0

**Published:** 2017-04-26

**Authors:** Stefanie Vandevijvere, Tara Mackenzie, Cliona Ni Mhurchu

**Affiliations:** 1 School of Population Health, The University of Auckland, Private bag 92019, Glen Innes, New Zealand; 2 National Institute for Health Innovation, The University of Auckland, Auckland, New Zealand

**Keywords:** Consumer retail food environments, INFORMAS, Shelf length, Food variety, Food availability, Supermarkets, Validation

## Abstract

**Background:**

In-store availability of healthy and unhealthy foods may influence consumer purchases. Methods used to measure food availability, however, vary widely. A simple, valid, and reliable indicator to collect comparable data on in-store food availability is needed.

**Methods:**

Cumulative linear shelf length of and variety within 22 healthy and 28 unhealthy food groups, determined based on a comparison of three nutrient profiling systems, were measured in 15 New Zealand supermarkets. Inter-rater reliability was tested in one supermarket by a second researcher. The construct validity of five simple indicators of relative availability of healthy versus unhealthy foods was assessed against this ‘gold standard’.

**Results:**

Cumulative linear shelf length was a more sensitive and feasible measure of food availability than variety. Four out of five shelf length ratio indicators were significantly associated with the gold standard (ρ = 0.70–0.75). Based on a non-significant difference from the ‘gold standard’ (d = 0.053 ± 0.040) and feasibility, the ratio of cumulative linear shelf length of fresh and frozen fruits and vegetables versus soft and energy drinks, crisps and snacks, sweet biscuits and confectionery performed best for use in New Zealand supermarkets.

**Conclusions:**

Four out of the five shelf length ratio indicators of the relative availability of healthy versus unhealthy foods in-store tested could be used for future research and monitoring, but additional validation studies in other settings and countries are recommended. Consistent use of those shelf length ratio indicators could enhance comparability of supermarket food availability between studies, and help inform policies to create healthy consumer food retail environments.

**Electronic supplementary material:**

The online version of this article (doi:10.1186/s12966-017-0512-0) contains supplementary material, which is available to authorized users.

## Background

Overweight and obesity are increasing in many countries globally [1]. A main driver of obesity is unhealthy food environments [2]. Individual behaviours are difficult to change, and interventions targeted at the individual have shown limited effectiveness at a high cost [3]. In contrast, structural interventions on environments can be cost saving, but are more challenging to implement.

Glanz defines the consumer food environment as that which customers encounter when buying food, including the cost, quality, and availability of food [4]. There is a growing body of research on consumer retail food environments, which are considered influential on food purchases, dietary behaviours and associated health outcomes [5, 6]. The relative availability of healthy versus unhealthy foods in-store is an important feature of consumer retail food environments. Systematic reviews evaluating associations between consumer retail food environments and dietary habits have however shown mixed results to date [5–7], due to the large heterogeneity in methods used [4, 5, 7].

There are six different ways in-store food availability has been measured in previous studies: 1) presence or absence, 2) linear shelf length, 3) proportion of shelf space, 4) shelf surface area, 5) number of displays and 6) variety (see Additional file 1: references). Some studies also measure shelf height; however, this increases the complexity of data collection. The way variety is measured differs between studies, with some excluding, or including, size, brand, and cultivars (for fresh produce, e.g. royal gala versus Braeburn apples) as different products in variety counts.

Previous studies measuring food availability in-store have often used predetermined food groups and categorized these as healthy or unhealthy. Food groups included have been selected based on various reasons, such as: commonly eaten [8–19], selected by experts [8, 9, 11, 12, 14, 17, 20, 21], part of a healthy diet in the region [8–17], contribution to chronic disease or caloric intake [14, 21–26], or selected in previous studies [8, 9, 11, 12, 14, 17, 20, 21, 27].

Variability in methods previously used to measure food availability has led to inconsistent and conflicting evidence on the effects of consumer retail food environments on purchasing behaviours and diet quality [28, 29]. The International Network for Food and Obesity/non-communicable diseases Research, Monitoring and Action Support (INFORMAS) is developing methods to measure and benchmark food environments among countries internationally [30]. The INFORMAS retail module outlines an evidence-based framework for global monitoring of both community and consumer retail food environments [5, 30]. The latter includes measures such as the relative availability, prominence and promotion of healthy versus unhealthy foods in-store. Development of a simple, reliable, and valid indicator of the relative availability of healthy versus unhealthy foods in-store would improve feasibility, allow for comparison between countries and studies, and could be used in further research to examine the relationships between consumer retail food environments and purchasing or dietary behaviors [5].

Therefore, the purpose of this study is to validate a set of simple indicators for measuring relative availability of healthy versus unhealthy foods in-store.

## Methods

Ethics approval for this study was obtained from the University of Auckland Human Participants Ethics Committee (reference number 012330). The study adhered to the principles within the Declaration of Helsinki. No written informed consent from store managers was required but a letter explaining the study was prepared for store managers as a matter of courtesy. Upon entrance of supermarkets, the study methods were explained to the store manager before starting data collection.

For the purposes of this study, the ‘gold standard’ is defined as the ratio of total availability of healthy foods versus total availability of unhealthy foods in-store. The simple indicators are ratios of availability of a selection of healthy food groups versus availability of a selection of unhealthy food groups in-store. Three nutrient profiling systems were applied to a database of packaged foods available in New Zealand supermarkets and compared to select the healthiest (*n* = 22) and unhealthiest (*n* = 28) food categories for inclusion as part of the ‘gold standard’. The shelf length and variety of these 50 food categories were measured in 15 New Zealand supermarkets across three different chains. The construct validity of five different simple indicators for the relative availability of healthy versus unhealthy foods in supermarkets, selected based on literature, was tested against the ‘gold standard’. Inter-rater reliability was tested in one supermarket by a second researcher.

### Selection of food groups to be included in the ‘gold standard’

Nutritrack is a database comprising food composition data on all packaged food products for sale in four major supermarket chains in New Zealand. Three nutrient profiling models were used to objectively determine which combinations of healthy and unhealthy food groups to include as part of the ‘gold standard’: the World Health Organisation (WHO) Europe nutrient profile model [31], the New Zealand Ministry of Health (MOH) Food and Beverage Classification System [32], and the Health Star Ratings system (HSR) [33]. Products with = <1.5 stars were considered unhealthy foods and those with > = 4 stars were considered healthy foods according to the HSR system for the purposes of this study. A total of 13,093 packaged food products were analysed in 2014 and detailed results of the comparison of those nutrient profiling systems have been published previously [34]. To be included as part of the gold standard, food groups needed to have at least 50% of their products classified as either healthy or unhealthy according to all three nutrient profiling systems. In addition, since specific anomalies (e.g. products getting more or less stars than expected) have been identified with the current HSR system [35], food groups that included 80% or more healthy or unhealthy products according to both the WHO and MOH systems were also included in the ‘gold standard’ (higher threshold of 80% since only 2 nutrient profiling systems compared) (Table 1). Sauces, edible oils and spreads are not included in the MOH system. As to not exclude them from this study, those that included 80% or more healthy or unhealthy products according to the WHO system were included in the ‘gold standard’. The final list of food groups included in the ‘gold standard’ (Additional file 2: Table S1), based on the comparison of the three nutrient profiling systems, was slightly adjusted after piloting the measurements in two supermarkets, and included 22 healthy and 28 unhealthy food groups, largely in line with the food groups commonly measured in previous research on food availability in-store.

**Table 1:** Criteria for food groups to be included as part of the healthy or unhealthy food groups in the gold standard

Nutrient Profiling System	Healthy food groups	Unhealthy food groups
All three systems	>50% of products in category meet healthy criteria	>50% of products in category meet unhealthy criteria
OR		
WHO and MoH systems	>80% of products in category met healthy criteria	>80% of products in category meet unhealthy criteria
OR		
Sauces and spreads	>80% of products in category meet WHO healthy criteria	>80% of products in category meet WHO unhealthy criteria

*HSR* health star ratings, *MOH* Ministry of Health, *WHO* World Health Organization

### Selection of simple indicators to be tested from literature

Availability ratios of all possible healthy versus unhealthy food group combinations from the original 50 food categories in the ‘gold standard’ (Additional file 2: Table S1) would be an unrealistic amount of possibilities to test. Therefore, five simple indicators (Table 2) were selected from previous literature looking at availability of healthy and unhealthy foods in-store, and which included not too many foods for the simple indicators to become impractical. All selected indicators comprised a subset of healthy and unhealthy food groups as included in the ‘gold standard’.

**Table 2:** Selected simple indicators for measuring the relative availability of healthy versus unhealthy foods in supermarkets

Indicator	Healthy and unhealthy food groups included	Rationale
1	fresh and frozen fruit and vegetables (including packaged) vs. soft drinks, crisps and snacks, sweet biscuits, cakes and slices, confectionery	Based on food categories used in previous studies by Farley et al 2009 [38], Rose et al 2009 [23], Miller 2012 [24], and Bodor et al 2013 [39]. These studies did include canned fruit and vegetables but these were not included in the ‘gold standard’ in this study, so excluded.
2	fresh fruit and vegetables (including packaged) vs. soft drinks and energy drinks	These food groups represent the most commonly included healthy and unhealthy food categories in previous research on food availability in-store
3	fresh fruit and vegetables (including packaged) vs. soft drinks, crisps and snacks, confectionery	The food groups in this indicator are those used in the Australian study by Cameron et al 2013 [40]. Fresh fruit and vegetables were used by 15 previous studies as the only healthy food items measured. Soft drinks, crisps, and confectionery were the three most commonly measured unhealthy foods in previous studies.
4	fresh and frozen fruit and vegetables (including packaged) vs. soft drinks, energy drinks, crisps and snacks, sweet biscuits, confectionery	Similar to indicator 1 but excludes the cakes and slices to improve feasibility of measurements in-store.
5	frozen fruit and vegetables vs. soft drinks, energy drinks, crisps and snacks	The included food categories were all common inclusions as healthy or unhealthy foods in previous studies. Excludes fresh fruit and vegetables as these products require more complex measurements to be performed in-store (e.g. measuring bins, no clear shelves).

### Selection of stores

This study focused on supermarkets as the dominant grocery retailers in New Zealand from which New Zealanders mainly purchase their foods. The two leading supermarket retailers in New Zealand are Progressive Enterprises and FoodStuffs, which together make up more than 90% of the market share according to Euromonitor [36]. Foodstuffs owns the supermarket chains Pak’nSave and New World, which have 53 and 139 stores across New Zealand and a 54% value share. Progressive Enterprises owns the chain Countdown, which has 183 supermarkets in New Zealand and a 38% value share. A convenience sample of 15 supermarkets in Auckland, New Zealand, was selected, across the three major chains (Countdown, Pak’nSave, New World) of medium to large supermarkets, defined as supermarkets with three or more cash registers. The areas in which these supermarkets were located covered a range of different socioeconomic deprivation levels. One supermarket manager declined participation because he didn’t want pictures to be taken within the supermarket. This supermarket was replaced by another one from the same chain.

### Data collection

Data collection was carried out between October 2015 and January 2016 (excluding December). The cumulative linear shelf length of and variety within the 50 different food groups included in the ‘gold standard’ (and simultaneously for the subset of food groups included within each of the five indicators) were measured by supermarket area.

Supermarket areas are based on the nine locations used in the validated Go Promo tool [37], and include: outside, entrance, endcaps front, endcaps back, aisles, edge, islands, checkouts side and checkouts end. The outside area was excluded for obvious reasons.

Linear shelf length of the different food groups was measured in meters using a laser instrument (Bosch PLR50, with measurement accuracy to 2 mm) either along the shelf or along the floor in front of the shelf. The number of shelves (of equal measured length) on which the food category was displayed was also recorded and multiplied by the linear shelf length to obtain the cumulative shelf length for each food category. If shelf length for a particular food category was different across different shelves, the shelf length was measured and recorded for each shelf separately and then summed to produce a total shelf length. For shelving units that did not have a physical shelf (e.g. units with hanging confectionery), rows of hanging products were counted as a single shelf. Displays that contained multiple rows of different products (for example. deli meats or dividers between frozen food) were also be counted as multiple ‘shelves’ in this way. Measurement of islands/freestanding bins was performed by measuring the exposed sides from which customers could pick products, as consistent with previous studies [23, 38]. For round freestanding bins, the diameter was measured and circumference calculated using 2πr. A paper data collection sheet was used to record the shelf length measurements.

In addition to measuring shelf length, photos of shelf sections including the different products were taken, including details on front-of-pack and price tags with names of products to aid in identifying different varieties. The photos were then sorted into the different healthy and unhealthy food categories and the number of product varieties in each was counted using a hand held counter. Variety was defined as the total number of different food products available in the supermarket within a certain food category. Two different counts of variety were used:

1. Variety including different sizes, flavours, and variations such as fair trade/organic, countries of origin (e.g. Australian versus American oranges), and cultivars (e.g. royal gala versus Braeburn apples) as different products.
2. Same as 1. but excluding different sizes of the same product, i.e. different sizes are counted only once.

All measurements were conducted by a single researcher following a standard protocol. The researcher was trained (including on the inclusions and exclusions for each of the 50 food groups to be measured) and pilot tested the protocol in two supermarkets before starting actual data collection. For some of the food groups included, shelf length was not easily measurable in the supermarket, as food items in those groups were not placed together on the shelves. For this reason, some food groups were either further divided or combined into different sub groups that were more feasible for measurement after the pilot test. For the purposes of calculating inter-rater reliability, one supermarket was assessed by a second researcher on the same day. Cumulative shelf length of and variety within all 50 food categories were compared between both researchers.

### Statistical analysis

Data was analysed using IBM SPSS statistical software version 23 (IBM Corp, Armonk, NY, USA 2015). Inter-rater reliability was tested using intra-class correlations (ICCs). In addition, the absolute difference in cumulative shelf length and variety counts between the two researchers was calculated, as well as the difference as a percentage of the average shelf length/variety.

The construct validity of the five simple indicators for the relative availability of healthy versus unhealthy foods in supermarkets was tested through: 1) a Wilcoxon signed rank test to assess the difference between each simple indicator and the ‘gold standard’, and 2) Spearman rank correlation coefficients to assess associations between the ‘gold standard’ and each simple indicator.

## Results

Table 3 shows the average cumulative linear shelf length of food groups categorised as healthy and unhealthy, as well as the ratio of cumulative linear shelf length of healthy vs. unhealthy foods. In addition, the number of varieties in each category is also presented. A consistently low ratio of healthy to unhealthy products is evident across all three measures of food availability and ranges from 0.2 to 0.4. There is also considerable variation across the different supermarket chains, especially for shelf length ratios, although the sample size for such comparison is low. When excluding check-outs and end-of-aisle endcaps from the measurements of cumulative shelf length (products in these locations tend to change more quickly than in other supermarket locations and check-outs are often harder to measure in view of customers lining up), the ratio of cumulative linear shelf length for healthy vs. unhealthy foods increases. Shelf length ratio did not appear to be related to variety ratio.

**Table 3:** Cumulative linear shelf length of and variety within healthy and unhealthy foods (gold standard) in supermarkets

	Chain A (*n* = 5 stores)		Chain B (*n* = 5 stores)		Chain C (*n* = 5 stores)	
	Average	Standard Deviation	Average	Standard Deviation	Average	Standard Deviation
Cumulative linear shelf length						
TOTAL HEALTHY shelf length (m)	437.0	142.7	230.3	40.3	335.1	60.2
TOTAL UNHEALTHY shelf length (m)	1151.0	308.6	909.0	218.3	1907.5	400.5
Ratio total healthy: total unhealthy shelf length	0.38	0.06	0.27	0.11	0.18	0.02
Cumulative linear shelf length (excluding endcaps and check-outs)						
TOTAL HEALTHY shelf length (m)	422.8	134.7	221.0	32.6	328.7	59.0
TOTAL UNHEALTHY shelf length (m)	998.4	299.5	791.2	195.1	1666.6	337.1
Ratio total healthy: total unhealthy shelf length	0.43	0.06	0.30	0.12	0.20	0.02
Variety 1 (including different package sizes as different products)						
TOTAL HEALTHY varieties	675.0	146.6	550.0	23.9	712.6	54.1
TOTAL UNHEALTHY varieties	2842.6	516.9	2093.6	61.8	2571.2	348.9
Ratio total healthy: total unhealthy varieties	0.24	0.02	0.26	0.01	0.28	0.03
Variety 2 (excluding different package sizes as different products)						
TOTAL HEALTHY varieties	578.6	134.0	488.4	21.3	633.2	58.5
TOTAL UNHEALTHY varieties	2397.0	465.8	1799.0	65.0	2157.6	316.7
Ratio total healthy: total unhealthy varieties	0.24	0.02	0.27	0.01	0.30	0.03

All three measures (shelf length, variety measures 1 and 2) showed very good inter-rater reliability with all three ICCs = 0.99, showing near perfect agreement. The largest differences in cumulative linear shelf length measured between both researchers were found for Asian sauces, coconut cream and milk, cereal bars, sugars, reduced fat powdered milks, plain noodles, plain couscous, breakfast biscuits, chilled seafood, dried vegetables and legumes, and unsalted nuts, representing 11 of the 50 food categories measured (data not shown).

Table 4 shows the results of the comparison between the five different simple indicators and the ‘gold standard’ of relative availability of total healthy versus total unhealthy foods in supermarkets. Shelf length ratio indicators 1,2,3,4 are all significantly associated with the gold standard (*ρ* = 0.70–0.75). Both shelf length ratio indicators 1 and 4 show a non-significant difference (*p* > 0.05) compared to the ‘gold standard’ as well as a significant association with the gold standard (*p* < 0.05). In regards to the variety ratios, the indicators perform better when different sizes are not included in the variety measure as different products. Variety ratio indicators 1, 2, 4 and 5 are all significantly associated with the gold standard (*ρ* = 0.56–0.75). Only indicator 1 and 5 are both significantly associated with the gold standard, as well as not significantly different from the gold standard (Table 4).

**Table 4:** Difference and correlation between the different simple indicators and the ‘gold standard’ for measuring relative availability of healthy versus unhealthy foods in supermarkets

Measure	Result	Indicator 1	Indicator 2	Indicator 3	Indicator 4	Indicator 5
		fresh and frozen fruit and vegetables: soft drinks, crisps, sweet biscuits, cakes and slices, confectionery	fresh fruit and vegetables: soft drinks, energy drinks	fresh fruit and vegetables: soft drinks, crisps, confectionery	fresh and frozen fruit and vegetables: soft drinks, energy drinks, crisps, sweet biscuits, confectionery	frozen fruit and vegetables: soft drinks, energy drinks, crisps and snacks
Shelf length	Average ratio	0.28 ± 0.07	1.24 ± 0.38	0.35 ± 0.10	0.29 ± 0.08	0.07 ± 0.04
	Range (min-max) of ratio	0.17–0.45	0.65–1.88	0.18–0.53	0.16–0.48	0.03-0.19
	Average difference from ‘gold standard’	0.057 ± 0.040	0.962 ± 0.317	0.078 ± 0.074	0.053 ± 0.040	0.207 ± 0.114
	z-score (Wilcoxon)	-0.057	-3.408	-3.010	-0.795	-3.408
	*p*-value (Wilcoxon)	0.955	0.001	0.003	0.427	0.001
	Correlation (spearman)	0.696	0.743	0.750	0.750	0.079
	*p*-value	0.004	0.002	0.001	0.001	0.781
Variety (including different sizes)	Average ratio	0.23 ± 0.03	0.85 ± 0.17	0.23 ± 0.04	0.25 ± 0.03	0.19 ± 0.05
	Range (min-max) of ratio	0.17–0.30	0.59–1.21	0.16–0.31	0.18–0.29	0.11–0.26
	Average difference from ‘gold standard’	0.028 ± 0.0154	-0.586 ± 0.149	0.043 ± 0.016	0.020 ± 0.011	0.069 ± 0.039
	z-score (Wilcoxon)	-3.181	-3.408	-2.385	-1.874	-3.351
	*p*-value (Wilcoxon)	0.001	0.001	0.017	0.061	0.001
	Correlation (spearman)	0.311	0.632	0.332	0.507	0.404
	*p*-value	0.260	0.011	0.226	0.054	0.136
Variety (excluding different sizes)	Average ratio	0.27 ± 0.03	1.46 ± 0.39	0.28 ± 0.05	0.29 ± 0.03	0.27 ± 0.05
	Range (min-max) of ratio	0.19–0.34	0.77–2.29	0.19–0.37	0.22–0.34	0.18–0.34
	Average difference from ‘gold standard’	0.021 ± 0.014	1.193 ± 0.361	0.039 ± 0.030	0.029 ± 0.026	0.028 ± 0.030
	z–score (Wilcoxon)	-1.136	-3.408	-0.057	-2.897	-0.454
	*p*-value (Wilcoxon)	0.256	0.001	0.955	0.004	0.650
	Correlation (spearman)	0.557	0.754	0.318	0.564	0.686
	*p*-value	0.031	0.001	0.248	0.028	0.005

The differences between the indicators and the gold standard also showed some variation among different supermarket chains. Shelf length ratio indicators 1 and 4 showed the smallest difference from the ‘gold standard’ across all three brands. Indicator 2 showed the largest differences with the ‘gold standard’ across all three chains and all three measures (shelf length, variety 1 and variety 2) (data not shown).

## Discussion

This study validated five indicators of relative availability of healthy versus unhealthy foods in-store against a gold standard. Healthy and unhealthy food availability were assessed by measuring both cumulative linear shelf length, as well as variety. These are two different concepts, as shelf length indicates the shelf space taken up by certain food groups regardless of the variety of foods within those food groups, while variety indicates the different choices available within each food group, regardless of the shelf space taken up by those food groups.

Cumulative linear shelf length was shown to be a more sensitive measure for food availability in supermarkets than variety. The measures of shelf length are also less time consuming than those for variety, and more acceptable to supermarket retailers who expressed some concerns about taking pictures of shelf sections in-store. A limitation of the shelf length ratio measure is that some food products are not placed on a physical shelf (e.g. hanging confectionary or fruit in freestanding bins) and methods were slightly adapted to be able to measure shelf length for those products.

The variety ratios of healthy versus unhealthy food products differed less dramatically between store types compared to shelf length ratios of healthy versus unhealthy foods. The variety measures would be less practical in larger studies due to the time burden and difficulties around permissions for taking pictures in-store.

To measure validity of the five shelf length ratio indicators against a gold standard, we evaluated both correlations between indicators and the ‘gold standard’, as well as the difference between indicators and the ‘gold standard’. For New Zealand supermarkets specifically, the ‘gold standard’ includes all healthy and unhealthy foods in-store and thus measures the relative availability of healthy versus unhealthy foods in-store adequately. Therefore, for New Zealand supermarkets, shelf length indicators 1 and 4 show the most potential as simple and valid indicators of in-store availability of healthy versus unhealthy foods. These indicators show a non-significant difference from the ‘gold standard’ indicator for shelf length as well as a significant correlation with the gold standard. Both of these indicators would be much faster and easier to measure in-store food availability than the ‘gold standard’ and still be similar to the ‘gold standard’ ratio of healthy versus unhealthy foods in each store. Indicator 4 is further preferred in view of the difficulty of measuring cakes and slices in-store (as they are found in many different locations across the store) and thus has a higher feasibility for monitoring. Therefore, indicator 4 (cumulative linear shelf length of fresh and frozen fruit and vegetables versus soft drinks and energy drinks, crisps and snacks, sweet biscuits and confectionery) would be the best indicator to use in future research and monitoring as a valid indicator of the availability of healthy and unhealthy foods in New Zealand supermarkets.

However, for other countries, settings and contexts, four out of the five shelf length ratio indicators could be used in future research and monitoring since they were all significantly correlated to the gold standard and correlations were very similar. The difference between the indicators and the gold standard, as measured in this study, is less relevant in this case since in other countries and contexts the foods available in the food groups as part of the indicators may differ, as well as the shelf length they take up in the supermarkets. In addition, in other contexts researchers may want to use different nutrient profiling systems to determine the healthy and unhealthy food groups as part of the gold standard. Therefore, in other countries and contexts, additional validation studies using those four shelf length ratio indicators are recommended.

The findings of this study are largely consistent with previous research using similar indicator food groups that showed shelf length dedicated to unhealthy food to be higher than the shelf length allocated to healthy foods in supermarkets [23, 38]. Using the ‘gold standard’ measurement, the ratios calculated in this study varied between 0.18 and 0.38, depending on supermarket chain.

### Feasibility

Performing data collection for the ‘gold standard’ presented multiple challenges and took on average eight hours to complete per supermarket, including shelf length measurements, photo taking, and variety counts afterwards using the photos. Management of pictures and counting of variety after data collection in the supermarkets took up to five hours, as on average 800 photos were taken at each supermarket and an average of 2500 unhealthy and 650 healthy varieties counted in each store. Measuring the gold standard was time consuming as some products were found in many locations around the supermarket on small sections of shelf, which meant many measurements were made to collect the total cumulative linear shelf length of the food group. This was particularly the case for food groups such as unsalted nuts, dried vegetables and legumes, confectionary, cakes muffins and pastries, table sauces, and meal based sauces and marinades. It is important for data collectors to have good knowledge on inclusion and exclusion criteria for foods within each of the 50 food groups included as part of the gold standard. In addition, the pictures need to be of sufficient resolution to easily read the product’s price tags, which were useful in distinguishing a product as a new variety or size (as they typically contained a detailed description and size of the product).

The indicator selected for New Zealand supermarkets based on the results from this study (cumulative linear shelf length of fresh and frozen fruit and vegetables versus soft drinks and energy drinks, crisps and snacks, sweet biscuits and confectionery) takes about one hour to measure in-store and no pictures need to be taken, which makes it a feasible measure for use in larger studies. Indicator 2 and 3 include even fewer food groups and may thus take even less time to measure in-store, while indicator 1 is a bit more time consuming due to the cakes and slices being located in many different places in-store.

### Strengths and limitations

This study is the first of its kind to validate a series of simple indicators for the relative availability of healthy versus unhealthy foods in-store to improve feasibility and standardization of monitoring efforts, such as those by INFORMAS [5]. We used relevant international literature and nutrient profiling systems to guide selection of food availability measures, food categories as part of the gold standard and simple indicators using different combinations of healthy and unhealthy food categories. This study provides valuable information about the construct validity and feasibility of a series of indicators for the relative availability of healthy versus unhealthy foods in-store for use in future research. The advantage of using a ratio rather than absolute measures is that it also minimizes the effects of store size, allowing for comparison between stores.

The study included a few limitations. The Nutritrack database and HSR nutrient profiling system only cover packaged foods with a Nutrition Information Panel, so assumptions had to be made based on packaged versions of foods for inclusion or exclusion (e.g. packaged fruit and vegetables). Another limitation of this study was that supermarkets measured in October-November already had many Christmas themed confectionery, cakes, and sweet biscuits. In addition, the supermarkets visited in the January period (when Christmas stock was expected to no longer be on shelves) still had some leftover Christmas period stock as well as some Easter confectionery themed products in store already. This may have influenced the shelf length and variety counts of unhealthy products from these supermarkets.

The study only included supermarkets and could be repeated to validate in-store measures for other types of outlets, especially in areas where there may not be large supermarkets available. For example, smaller convenience-type stores are more commonly found rurally and would be important to include as part of an assessment of in-store food availability. Other measures might however need to be developed for small stores, as they do not have the full range of products available such as in supermarkets.

### Implications for further research and monitoring

Four out of five shelf length ratio indicators of relative availability of healthy and unhealthy foods in supermarkets tested in this study can be used for future research and monitoring, but additional validation studies will be required in other settings, especially in low and middle income countries. For New Zealand specifically, the linear cumulative shelf length allocated to fresh and frozen fruit and vegetables versus soft drinks and energy drinks, crisps, sweet biscuits and confectionery provides the best indicator for the availability of healthy versus unhealthy foods in supermarkets. Use of this indicator would provide a valid, consistent tool to collect data in a large number of supermarkets and settings, to both monitor food availability and assess its effects on consumer purchases. When using the indicators in future research and monitoring, it is important to consider the frequency and period of measurements (seasonality, frequency of changes of products within stores), sampling of supermarkets and duration of data collection. Both the gold standard and the indicators included all locations in the supermarket, but it needs to be acknowledged that the foods in some locations (endcaps and checkouts) may change more quickly than in other supermarket locations (e.g. aisles). However, excluding these locations from the study is not recommended as 1) those are among the most prominent locations in the supermarket, 2) this would not represent an adequate measure of availability and 3) it is likely that, such as in this study, the ratios on the relative availability of healthy versus unhealthy foods would increase as unhealthy foods are predominant in those locations. The indicator, similar as the gold standard, showed large variety across stores and chains in New Zealand, which means sufficient supermarkets would need to be included in a sample to be representative of the healthiness of the in-store retail food environment at national or regional level.

## Conclusions

Four out of five shelf length ratio indicators tested in this study could be used for future research and monitoring, but additional testing and validation in other settings is recommended. Ratio of cumulative linear shelf length of fresh and frozen fruit and vegetables to soft drinks and energy drinks, crisps and snacks, sweet biscuits and confectionery provides a simple and feasible indicator of the in-store availability of healthy versus unhealthy foods in New Zealand supermarkets. Consistent use of those shelf length ratio indicators in future research and monitoring would enhance comparability of food availability between different countries and studies, and strengthen the research linking the in-store food environment with diet and obesity.

## Additional files

Additional file 1: Supplementary references of previous studies measuring food availability in-store. (PDF 146 kb)

Additional file 2: Table S1. List of the 22 healthy and 28 unhealthy food groups part of the ‘gold standard’ as derived from the comparison of three nutrient profiling systems. (DOCX 15.5 kb)

## Abbreviations

HSR: Health star rating
ICC: Intra-class correlations
INFORMAS: International network for food and obesity research, monitoring and action support
MOH: Ministry of health
WHO: World Health Organization
