# Supplementary material for: Indicators of the relative availability of healthy versus unhealthy foods in supermarkets: a validation study
Source: Int J Behav Nutr Phys Act. 2017 Apr 26;14:53. doi: 10.1186/s12966-017-0512-0 (PMC5405544; doi:10.1186/s12966-017-0512-0)
Supplement: Supplementary file 1 — Supplementary references of previous studies measuring food availability in-store. (PDF 146 kb) [file 12966_2017_512_MOESM1_ESM.pdf]

Additional file 1: references

1. Rimkus L, Powell LM, Zenk SN, Han E, Ohri-Vachaspati P, Pugach O, et al. Development and reliability testing of a food store observation form. J Nutr Educ Behav. 2013;45(6):540-8.
2. Farley TA, Rice J, Bodor JN, Cohen DA, Bluthenthal RN, Rose D. Measuring the food environment: shelf space of fruits, vegetables, and snack foods in stores. Journal of Urban Health. 2009;86(5):672-82.
3. Franco M, Diez-Roux AV, Nettleton JA, Lazo M, Brancati F, Caballero B, et al. Availability of healthy foods and dietary patterns: the Multi-Ethnic Study of Atherosclerosis. American Journal of Clinical Nutrition. 2009;89(3):897-904.
4. Olendzki BC, Procter-Grey E, Wedick NM, Patil V, Zheng H, Kane K, et al. Disparities in Access to Healthy and Unhealthy Foods in Central Massachusetts: Implications for Public Health Policy. Journal of the American College of Nutrition. 2015:1-9.
5. Sharkey JR, Dean WR, Nalty C. Convenience stores and the marketing of foods and beverages through product assortment. American Journal of Preventive Medicine. 2012;43(3 Suppl 2):S109-15.
6. Gloria CT, Steinhardt MA. Texas nutrition environment assessment of retail food stores (TxNEA-S): development and evaluation. Public Health Nutr. 2010;13(11):1764-72.
7. Martin KS, Ghosh D, Page M, Wolff M, McMinimee K, Zhang M. What role do local grocery stores play in urban food environments? A case study of Hartford-Connecticut. PLoS ONE. 2014;9(4):e94033.
8. Lee RE, Heinrich KM, Medina AV, Regan GR, Reese-Smith JY, Jokura Y, et al. A picture of the healthful food environment in two diverse urban cities. Environ Health Insights. 2010;4:49-60.
9. Hua J, Seto E, Li Y, Wang MC. Development and evaluation of a food environment survey in three urban environments of Kunming, China. BMC Public Health. 2014;14:235.
10. Black C, Ntani G, Kenny R, Tinati T, Jarman M, Lawrence W, et al. Variety and quality of healthy foods differ according to neighbourhood deprivation. Health Place. 2012;18(6):1292-9.
11. Block D, Kouba J. A comparison of the availability and affordability of a market basket in two communities in the Chicago area. Public Health Nutr. 2006;9(7):837-45.
12. Noseworthy BL, Williams PL, Blum I, Macleod M. The availability and relative cost of locally produced foods in grocery stores in Nova Scotia. Journal of Hunger and Environmental Nutrition. 2011;6(2):188-206.
13. Roos JA, Ruthven GA, Lombard MJ, McLachlan MH. Food availability and accessibility in the local food distribution system of a low-income, urban community in Worcester, in the Western Cape province. South African Journal of Clinical Nutrition. 2013;26(4):194-200.
14. Sloane DC, Diamant AL, Lewis LB, Yancey AK, Flynn G, Nascimento LM, et al. Improving the nutritional resource environment for healthy living through community-based participatory research. J Gen Intern Med. 2003;18(7):568-75.
15. Mojtahedi MC, Boblick P, Rimmer JH, Rowland JL, Jones RA, Braunschweig CL. Environmental barriers to and availability of healthy foods for people with mobility disabilities living in urban and suburban neighborhoods. Arch Phys Med Rehabil. 2008;89(11):2174-9.

16. Young CM, Batch BC, Svetkey LP. Effect of socioeconomic status on food availability and cost of the dietary approaches to stop hypertension (DASH) dietary pattern. Journal of Clinical Hypertension. 2008;10(8):603-11.
17. Lloyd S, Lawton J, Caraher M, Singh G, Horsley K, Mussa F. A tale of two localities: Healthy eating on a restricted income. Health Education Journal. 2011;70(1):48-56.
18. Gittelsohn J, Franceschini MCT, Rasooly IR, Ries AV, Ho LS, Pavlovich W, et al. Understanding the food environment in a low-income Urban setting: Implications for food store interventions. Journal of Hunger and Environmental Nutrition. 2008;2(2-3):33-50.
19. Horowitz CR, Colson KA, Hebert PL, Lancaster K. Barriers to buying healthy foods for people with diabetes: Evidence of environmental disparities. Am J Public Health. 2004;94(9):1549-54.
20. Izumi BT. Inter-rater reliability of a food store checklist to assess availability of healthier alternatives to the energy-dense snacks and beverages commonly consumed by children. 2014.
21. Johnson JS, Nobmann ED, Asay E. Factors related to fruit, vegetable and traditional food consumption which may affect health among Alaska Native People in Western Alaska. International journal of circumpolar health. 2012;71:17345.
22. Sauveplane-Stirling V, Crichton D, Tessier S, Parrett A, Garcia AL. The food retail environment and its use in a deprived, urban area of Scotland. Public Health. 2014;128(4):360-6.
23. Barratt J. The cost and availability of healthy food choices in southern Derbyshire. Journal of Human Nutrition and Dietetics. 1997;10(1):63-9.
24. Andreyeva T, Blumenthal DM, Schwartz MB, Long MW, Brownell KD. Availability and prices of foods across stores and neighborhoods: the case of New Haven, Connecticut. Health affairs. 2008;27(5):1381-8.
25. Andreyeva T, Luedicke J, Middleton AE, Long MW, Schwartz MB. Positive influence of the revised Special Supplemental Nutrition Program for Women, Infants, and Children food packages on access to healthy foods. Journal of the Academy of Nutrition & Dietetics. 2012;112(6):850-8.
26. Azuma AM, Gilliland S, Vallianatos M, Gottlieb R. Food access, availability, and affordability in 3 Los Angeles communities, Project CAFE, 2004-2006. Preventing chronic disease. 2010;7(2):A27.
27. Baker EA, Schootman M, Barnidge E, Kelly C. The role of race and poverty in access to foods that enable individuals to adhere to dietary guidelines. Preventing chronic disease. 2006;3(3):A76.
28. Ball K, Timperio A, Crawford D. Neighbourhood socioeconomic inequalities in food access and affordability. Health Place. 2009;15(2):578-85.
29. Bertoni AG, Foy CG, Hunter JC, Quandt SA, Vitolins MZ, Whitt-Glover MC. A multilevel assessment of barriers to adoption of dietary approaches to stop hypertension (DASH) among African Americans of low socioeconomic status. Journal of health care for the poor and underserved. 2011;22(4):1205-20.
30. Black C, Ntani G, Inskip H, Cooper C, Cummins S, Moon G, et al. Measuring the healthfulness of food retail stores: variations by store type and neighbourhood deprivation. Int. 2014;11:69.
31. Bovell-Benjamin AC, Hathorn CS, Ibrahim S, Gichuhi PN, Bromfield EM. Healthy food choices and physical activity opportunities in two contrasting Alabama cities. Health Place. 2009;15(2):429-38.
32. Burns CM, Gibbon P, Boak R, Baudinette S, Dunbar JA. Food cost and availability in a rural setting in Australia. Rural Remote Health. 2004;4(4):311.

33. Bustillos B, Sharkey JR, Anding J, McIntosh A. Availability of more healthful food alternatives in traditional, convenience, and nontraditional types of food stores in two rural Texas counties. Journal of the American Dietetic Association. 2009;109(5):883-9.
34. Cannuscio CC, Tappe K, Hillier A, Buttenheim A, Karpyn A, Glanz K. Urban food environments and residents' shopping behaviors. American Journal of Preventive Medicine. 2013;45(5):606-14.
35. Cavanaugh E, Mallya G, Brensinger C, Tierney A, Glanz K. Nutrition environments in corner stores in Philadelphia. Prev Med. 2013;56(2):149-51.
36. Chau CN, Zoellner JM, Hill JL. Availability of Healthy Food: Does Block Group Race and Income Matter? Journal of Hunger and Environmental Nutrition. 2013;8(1):22-38.
37. Chaudhari LS, Begay RC, Schulz LO. Fifteen years of change in the food environment in a rural Mexican community: the Maycoba project. Rural Remote Health. 2013;13(3):2404.
38. Cohen DA, Schoeff D, Farley TA, Bluthenthal R, Scribner R, Overton A. Reliability of a store observation tool in measuring availability of alcohol and selected foods. Journal of Urban Health. 2007;84(6):807-13.
39. Connell CL, Yadrick MK, Simpson P, Gossett J, McGee BB, Bogle ML. Food supply adequacy in the Lower Mississippi Delta. Journal of Nutrition Education & Behavior. 2007;39(2):77-83.
40. Cummins S, Smith DM, Aitken Z, Dawson J, Marshall D, Sparks L, et al. Neighbourhood deprivation and the price and availability of fruit and vegetables in Scotland. Journal of Human Nutrition & Dietetics. 2010;23(5):494-501.
41. Dale D, Darcey V, Quinlan JJ. Comparison of the availability of healthy foods in low income and high income neighborhoods in Philadelphia. Ethn Dis. 2009;19(2 SUPPL.3):S382-S3.
42. Donkin AJ, Dowler EA, Stevenson SJ, Turner SA. Mapping access to food in a deprived area: the development of price and availability indices. Public Health Nutrition. 2000;3(1):31-8.
43. Duran AC, Diez Roux AV, Latorre Mdo R, Jaime PC. Neighborhood socioeconomic characteristics and differences in the availability of healthy food stores and restaurants in Sao Paulo, Brazil. Health Place. 2013;23:39-47.
44. Edmonds J, Baranowski T, Baranowski J, Cullen KW, Myres D. Ecological and socioeconomic correlates of fruit, juice, and vegetable consumption among African-American boys. Prev Med. 2001;32(6):476-81.
45. Emond JA, Madanat HN, Ayala GX. Do Latino and non-Latino grocery stores differ in the availability and affordability of healthy food items in a low-income, metropolitan region? Public Health Nutrition. 2012;15(2):360-9.
46. Farley TA, Baker ET, Futrell L, Rice JC. The ubiquity of energy-dense snack foods: a national multicity study. American Journal of Public Health. 2010;100(2):306-11.
47. Gantner LA, Olson CM, Frongillo EA, Wells NM. Prevalence of nontraditional food stores and distance to healthy foods in a rural food environment. Journal of Hunger and Environmental Nutrition. 2011;6(3):279-93.
48. Gebauer H, Laska MN. Convenience stores surrounding urban schools: an assessment of healthy food availability, advertising, and product placement. Journal of Urban Health. 2011;88(4):616-22.
49. Ghirardelli A, Quinn V, Sugerman S. Reliability of a retail food store survey and development of an accompanying retail scoring system to communicate survey findings and identify vendors for healthful food and

marketing initiatives. Journal of Nutrition Education & Behavior. 2011;43(4 Suppl 2):S104-12.
50. Ghosh-Dastidar B, Cohen D, Hunter G, Zenk SN, Huang C, Beckman R, et al. Distance to store, food prices, and obesity in urban food deserts. American Journal of Preventive Medicine. 2014;47(5):587-95.
51. Giskes K, Van Lenthe FJ, Brug J, Mackenbach JP, Turrell G. Socioeconomic inequalities in food purchasing: the contribution of respondent-perceived and actual (objectively measured) price and availability of foods. Prev Med. 2007;45(1):41-8.
52. Glanz K, Sallis JF, Saelens BE, Frank LD. Nutrition Environment Measures Survey in stores (NEMS-S): development and evaluation. Am J Prev Med. 2007;32(4):282-9.
53. Grigsby-Toussaint DS, Zenk SN, Odoms-Young A, Ruggiero L, Moise I. Availability of commonly consumed and culturally specific fruits and vegetables in African-american and Latino neighborhoods. J Am Diet Assoc. 2010;110(5):746-52.
54. Gustafson A, Christian JW, Lewis S, Moore K, Jilcott S. Food venue choice, consumer food environment, but not food venue availability within daily travel patterns are associated with dietary intake among adults, Lexington Kentucky 2011. Nutrition journal. 2013;12:17.
55. Gustafson AA, Sharkey J, Samuel-Hodge CD, Jones-Smith J, Folds MC, Cai J, et al. Perceived and objective measures of the food store environment and the association with weight and diet among low-income women in North Carolina. Public Health Nutrition. 2011;14(6):1032-8.
56. Havens EK, Martin KS, Yan J, Dauser-Forrest D, Ferris AM. Federal nutrition program changes and healthy food availability. American Journal of Preventive Medicine. 2012;43(4):419-22.
57. Hillier A, McLaughlin J, Cannuscio CC, Chilton M, Krasny S, Karpyn A. The impact of WIC food package changes on access to healthful food in 2 low-income urban neighborhoods. Journal of Nutrition Education & Behavior. 2012;44(3):210-6.
58. Horacek TM, Erdman MB, Reznar MM, Olfert M, Brown-Esters ON, Kattelmann KK, et al. Evaluation of the food store environment on and near the campus of 15 postsecondary institutions. American Journal of Health Promotion. 2013;27(4):e81-90.
59. Hosler AS, Kammer JR. Trends of fruit and vegetable availability in neighbourhoods in Albany, NY, USA, 2003-2012. Public Health Nutrition. 2014;18(3):562-8.
60. Hosler AS, Rajulu DT, Fredrick BL, Ronsani AE. Assessing retail fruit and vegetable availability in urban and rural underserved communities. Preventing chronic disease. 2008;5(4):A123.
61. Hosler AS, Varadarajulu D, Ronsani AE, Fredrick BL, Fisher BD. Low-fat milk and high-fiber bread availability in food stores in urban and rural communities. Journal of Public Health Management & Practice. 2006;12(6):556-62.
62. Innes-Hughes C, Boylan S, King LA, Lobb E. Measuring the food environment in three rural towns in New South Wales, Australia. Health Promotion Journal of Australia. 2012;23(2):129-33.
63. Izumi BT, Zenk SN, Schulz AJ, Mentz GB, Sand SL, de Majo RF, et al. Inter-rater reliability of the food environment audit for diverse neighborhoods (FEAD-N). Journal of Urban Health. 2012;89(3):486-99.

64. Jetter KM, Cassady DL. The availability and cost of healthier food alternatives. American Journal of Preventive Medicine. 2006;30(1):38-44.
65. Kersten E, Laraia B, Kelly M, Adler N, Yen IH. Small food stores and availability of nutritious foods: a comparison of database and in-store measures, Northern California, 2009. Preventing chronic disease. 2012;9:E127.
66. Krukowski RA, West DS, Harvey-Berino J, Elaine Prewitt T. Neighborhood impact on healthy food availability and pricing in food stores. Journal of community health. 2010;35(3):315-20.
67. Laska MN, Borradaile KE, Tester J, Foster GD, Gittelsohn J. Healthy food availability in small urban food stores: a comparison of four US cities. Public Health Nutrition. 2010;13(7):1031-5.
68. Latham J, Moffat T. Determinants of variation in food cost and availability in two socioeconomically contrasting neighbourhoods of Hamilton, Ontario, Canada. Health Place. 2007;13(1):273-87.
69. Lee AJ, Darcy AM, Leonard D, Groos AD, Stubbs CO, Lowson SK, et al. Food availability, cost disparity and improvement in relation to accessibility and remoteness in Queensland. Aust N Z J Public Health. 2002;26(3):266-72.
70. Lee-Kwan SH, Kumar G, Ayscue P, Santos M, McGuire LC, Blanck HM, et al. Healthful food availability in stores and restaurants - american samoa, 2014. MMWR Morb Mortal Wkly Rep. 2015;64(10):276-8.
71. Leone AF, Rigby S, Betterley C, Park S, Kurtz H, Johnson MA, et al. Store type and demographic influence on the availability and price of healthful foods, Leon County, Florida, 2008. Preventing chronic disease. 2011;8(6).
72. Liese AD, Weis KE, Pluto D, Smith E, Lawson A. Food store types, availability, and cost of foods in a rural environment. Journal of the American Dietetic Association. 2007;107(11):1916-23.
73. Martins PA, Cremm EC, Leite FH, Maron LR, Scagliusi FB, Oliveira MA. Validation of an adapted version of the nutrition environment measurement tool for stores (NEMS-S) in an urban area of Brazil. Journal of Nutrition Education & Behavior. 2013;45(6):785-92.
74. Millichamp A, Gallegos D. Comparing the availability, price, variety and quality of fruits and vegetables across retail outlets and by area-level socio-economic position. Public Health Nutrition. 2013;16(1):171-8.
75. Minaker LM, Raine KD, Wild TC, Nykiforuk CI, Thompson ME, Frank LD. Construct validation of 4 food-environment assessment methods: adapting a multitrait-multimethod matrix approach for environmental measures. American journal of epidemiology. 2014;179(4):519-28.
76. Mooney C. Cost and availability of healthy food choices in a London health district. Journal of Human Nutrition and Dietetics. 1990;3(2):111-20.
77. Morland K, Filomena S. Disparities in the availability of fruits and vegetables between racially segregated urban neighbourhoods. Public Health Nutrition. 2007;10(12):1481-9.
78. O'Connell M, Buchwald DS, Duncan GE. Food access and cost in American Indian communities in Washington State. J Am Diet Assoc. 2011;111(9):1375-9.
79. Pereira RF, Sidebottom AC, Boucher JL, Lindberg R, Werner R. Assessing the food environment of a rural community: baseline findings from the heart of New Ulm project, Minnesota, 2010-2011. Preventing chronic disease. 2014;11:E36.
80. Pettinger C, Holdsworth M, Gerber M. 'All under one roof?' Differences in food availability and shopping patterns in Southern France and Central England. European journal of public health. 2008;18(2):109-14.

81. Rose D, O'Malley K, Dunaway LF, Bodor JN. The Influence of the WIC Food Package Changes on the Retail Food Environment in New Orleans. Journal of Nutrition Education and Behavior. 2014;46(3S):S38-S44.
82. Sharkey JR, Johnson CM, Dean WR. Food access and perceptions of the community and household food environment as correlates of fruit and vegetable intake among rural seniors. BMC geriatr. 2010;10:32.
83. Sheldon M, Gans KM, Tai R, George T, Lawson E, Pearlman DN. Availability, affordability, and accessibility of a healthful diet in a low-income community, Central Falls, Rhode Island, 2007-2008. Preventing chronic disease. 2010;7(2):A43.
84. Smith ML, Sunil TS, Salazar CI, Rafique S, Ory MG. Disparities of food availability and affordability within convenience stores in Bexar County, Texas. J Environ Public Health. 2013;2013:782756.
85. Temple NJ, Steyn NP, Fourie J, De Villiers A. Price and availability of healthy food: a study in rural South Africa. Nutrition. 2011;27(1):55-8.
86. Tester JM, Yen IH, Pallis LC, Laraia BA. Healthy food availability and participation in WIC (Special Supplemental Nutrition Program for Women, Infants, and Children) in food stores around lower- and higher-income elementary schools. Public Health Nutrition. 2010;14(6):960-4.
87. Thornton LE, Cameron AJ, McNaughton SA, Worsley A, Crawford DA. The availability of snack food displays that may trigger impulse purchases in Melbourne supermarkets. BMC Public Health. 2012;12:194.
88. Thornton LE, Crawford DA, Ball K. Neighbourhood-socioeconomic variation in women's diet: the role of nutrition environments. Eur J Clin Nutr. 2010;64(12):1423-32.
89. Wang J, Williams M, Rush E, Crook N, Forouhi NG, Simmons D. Mapping the availability and accessibility of healthy food in rural and urban New Zealand--Te Wai o Rona: Diabetes Prevention Strategy. Public Health Nutrition. 2010;13(7):1049-55.
90. Wedick NM, Ma Y, Olendzki BC, Procter-Gray E, Cheng J, Kane KJ, et al. Access to healthy food stores modifies effect of a dietary intervention. American Journal of Preventive Medicine. 2015;48(3):309-17.
91. Winkler E, Turrell G, Patterson C. Does living in a disadvantaged area entail limited opportunities to purchase fresh fruit and vegetables in terms of price, availability, and variety? Findings from the Brisbane Food Study. Health Place. 2006;12(4):741-8.
92. Zenk SN, Grigsby-Toussaint DS, Curry SJ, Berbaum M, Schneider L. Short-term temporal stability in observed retail food characteristics. Journal of Nutrition Education & Behavior. 2010;42(1):26-32.
93. Zenk SN, Powell LM, Rimkus L, Isgor Z, Barker DC, Ohri-Vachaspati P, et al. Relative and absolute availability of healthier food and beverage alternatives across communities in the United States. American Journal of Public Health. 2014;104(11):2170-8.
94. Zenk SN, Schulz AJ, Israel BA, James SA, Bao S, Wilson ML. Fruit and vegetable access differs by community racial composition and socioeconomic position in Detroit, Michigan. Ethn Dis. 2006;16(1):275-80.
95. Rose D, Hutchinson PL, Bodor JN, Swalm CM, Farley TA, Cohen DA, et al. Neighborhood food environments and Body Mass Index: the importance of in-store contents. American Journal of Preventive Medicine. 2009;37(3):214-9.
96. Bodor JN, Rose D, Farley TA, Swalm C, Scott SK. Neighbourhood fruit and vegetable availability and consumption: the role of small food stores in an urban environment. Public Health Nutr. 2008;11(4):413-20.

97. Cameron AJ, Thornton LE, McNaughton SA, Crawford D. Variation in supermarket exposure to energy-dense snack foods by socio-economic position. Public Health Nutr. 2013;16(7):1178-85.
98. Thornton LE, Cameron AJ, McNaughton SA, Waterlander WE, Sodergren M, Svastisalee C, et al. Does the availability of snack foods in supermarkets vary internationally? International Journal of Behavioral Nutrition and Physical Activity. 2013;10.
99. Fisher BD, Strogatz DS. Community measures of low-fat milk consumption: Comparing store shelves with households. American Journal of Public Health. 1999;89(2):235-7.
100. Wechsler H, Basch CE, Zybert P, Lantigua R, Shea S. The availability of low-fat milk in an inner-city Latino community: implications for nutrition education. American Journal of Public Health. 1995;85(12):1690-2.
101. Bodor JN, Hutchinson PL, Rose D. Car ownership and the association between fruit and vegetable availability and diet. Prev Med. 2013;57(6):903-5.
102. Caldwell EM, Miller Kobayashi M, DuBow WM, Wytinck SM. Perceived access to fruits and vegetables associated with increased consumption. Public health nutrition. 2009;12(10):1743-50.
103. Cavanaugh E, Green S, Mallya G, Tierney A, Brensinger C, Glanz K. Changes in food and beverage environments after an urban corner store intervention. Prev Med. 2014;65:7-12.
104. Cheadle A, Psaty BM, Curry S, Wagner E, Diehr P, Koepsell T, et al. Community-level comparisons between the grocery store environment and individual dietary practices. Prev Med. 1991;20(2):250-61.
105. Cheadle A, Psaty B, Wagner E, Diehr P, Koepsell T, Curry S, et al. Evaluating community-based nutrition programs: assessing the reliability of a survey of grocery store product displays. Am J Public Health. 1990;80(6):709-11.
106. Bertrand L, Therien F, Cloutier MS. Measuring and mapping disparities in access to fresh fruits and vegetables in Montreal. Canadian Journal of Public Health Revue Canadienne de Sante Publique. 2008;99(1):6-11.
107. Pouliot N, Hamelin AM. Disparities in fruit and vegetable supply: a potential health concern in the greater Quebec City area. Public Health Nutrition. 2009;12(11):2051-9.
108. Vinkeles Melchers NVS, Gomez M, Colagiuri R. Do socio-economic factors influence supermarket content and shoppers' purchases? Health Promotion Journal of Australia. 2009;20(3):241-6.
109. Miller C, Bodor JN, Rose D. Measuring the food environment: a systematic technique for characterizing food stores using display counts. Journal of environmental and public health. 2012;2012:707860.
110. Chacon V, Letona P, Barnoya J. Child-oriented marketing techniques in snack food packages in Guatemala. BMC Public Health. 2013;13:967.
111. Kipke MD, Iverson E, Moore D, Booker C, Ruelas V, Peters AL, et al. Food and Park Environments: Neighborhood-level Risks for Childhood Obesity in East Los Angeles. Journal of Adolescent Health. 2007;40(4):325-33.
112. Lear SA, Gasevic D, Schuurman N. Association of supermarket characteristics with the body mass index of their shoppers. Nutrition journal. 2013;12:117.
113. Stroebele N, Dietze P, Tinnemann P, Willich SN. Assessing the variety and pricing of selected foods in socioeconomically disparate districts of Berlin, Germany. J Public Health (Oxf). 2011;19(1):23-8.
