# Supplementary material for: Indicators of the relative availability of healthy versus unhealthy foods in supermarkets: a validation study
Source: Int J Behav Nutr Phys Act. 2017 Apr 26;14:53. doi: 10.1186/s12966-017-0512-0 (PMC5405544; doi:10.1186/s12966-017-0512-0)
Supplement: Supplementary file 2 — List of the 22 healthy and 28 unhealthy food groups part of the ‘gold standard’ as derived from the comparison of three nutrient profiling systems. (DOCX 15.5 kb) [file 12966_2017_512_MOESM2_ESM.docx]

# Additional file 2: Table S1

| **Healthy products (n=22)** | **Unhealthy products (n=28)** |
| --- | --- |
| Frozen fruit | Sweet biscuits |
| Frozen vegetables | Confectionary (including chocolate) |
| Unsalted nuts | Cream |
| Dried vegetables and legumes | Butter |
| Fresh fruit (including packaged) | Salami and cured meats |
| Fresh vegetables (including packaged) | Soft drinks |
| Chilled seafood | Energy and electrolyte drinks |
| Uncoated frozen fish | Crisps and snacks |
| Eggs | Sugars and related products (dessert additions, sugar based products (curds, choc chips, treacle, etc.), icing, honey, dessert toppings) |
| Plain rice | Cakes, muffins and pastries |
| Frozen uncoated meat | Ice cream and edible ices |
| Polenta | Packet pasta |
| Quinoa | Prepared sandwiches |
| Plain oats | Fruit and vegetable juices |
| Breakfast biscuits | Beverage mixes and cordials |
| Plain couscous | Cereal bars and fruit bars |
| Plain noodles | Condensed milk |
| Plain dry pasta | Plain dairy milk – full cream |
| Still water | Plain powdered milk – full cream |
| Cooking oil spray | Plain powdered milk – whole |
| Reduced fat dairy milks | Coconut cream + milk |
| Reduced fat powdered milk | Frozen meat with pastry |
|  | Asian sauces |
|  | Table sauces |
|  | Other spreads |
|  | Meal-based sauces and marinades |
|  | Mayonnaise |
|  | Honey |
